# Exosomes derived from GDNF-modified human adipose mesenchymal stem cells ameliorate peritubular capillary loss in tubulointerstitial fibrosis by activating the SIRT1/eNOS signaling pathway

**DOI:** 10.7150/thno.43315

**Published:** 2020-07-25

**Authors:** Lu Chen, Yanping Wang, Shulin Li, Bangjie Zuo, Xiangyu Zhang, Fengzhen Wang, Dong Sun

**Affiliations:** 1 Department of Nephrology, Affiliated Hospital of Xuzhou Medical University, Xuzhou, China.; 2 Department of Internal Medicine and Diagnostics, Xuzhou Medical University, Xuzhou, China.; 3 Department of Rheumatology, Ningbo Medical Treatment Center Li Huili Hospital, Ningbo, China.; 4 Department of Pharmaceutics, Affiliated Hospital of Xuzhou Medical University, Xuzhou, China.

**Keywords:** Glial cell line-derived neurotrophic factor, Adipose tissue-derived mesenchymal stem cell, Exosome, Peritubular capillary, Chronic kidney disease

## Abstract

Mesenchymal stem cells (MSCs) have emerged as ideal cell-based therapeutic candidates for the structural and functional restoration of the diseased kidney. Glial cell line-derived neurotrophic factor (GDNF) has been demonstrated to promote the therapeutic effect of MSCs on ameliorating renal injury. The mechanism may involve the transfer of endogenous molecules via paracrine factors to salvage injured cells, but these factors remain unknown.

**Methods:** GDNF was transfected into human adipose mesenchymal stem cells *via* a lentiviral transfection system, and exosomes were isolated (GDNF-AMSC-exos). Using the unilateral ureteral obstruction (UUO) mouse model and human umbilical vein endothelial cells (HUVECs) against hypoxia/serum deprivation (H/SD) injury models, we investigated whether GDNF-AMSC-exos ameliorate peritubular capillary (PTC) loss in tubulointerstitial fibrosis and whether this effect is mediated by the Sirtuin 1 (SIRT1) signaling pathway. Additionally, by using SIRT1 activators or siRNAs, the roles of the candidate mRNA and its downstream gene in GDNF-AMSC-exo-induced regulation of endothelial cell function were assessed. PTC characteristics were detected by fluorescent microangiography (FMA) and analyzed by the MATLAB software.

**Results:** The green fluorescent PKH67-labeled exosomes were visualized in the UUO kidneys and colocalized with CD81. GDNF-AMSC-exos significantly decreased PTC rarefaction and renal fibrosis scores in mice with UUO. *In vitro* studies revealed that GDNF-AMSC-exos exerted cytoprotective effects on HUVECs against H/SD injury by stimulating migration and angiogenesis as well as conferring apoptosis resistance. Mechanistically, GDNF-AMSC-exos enhanced SIRT1 signaling, which was accompanied by increased levels of phosphorylated endothelial nitric oxide synthase (p-eNOS). We also confirmed the SIRT1-eNOS interaction in HUVECs by immunoprecipitation. Furthermore, we observed a correlation of the PTC number with the SIRT1 expression level in the kidney *in vivo*.

**Conclusion:** Our study unveiled a mechanism by which exosomes ameliorate renal fibrosis: GDNF-AMSC-exos may activate an angiogenesis program in surviving PTCs after injury by activating the SIRT1/eNOS signaling pathway.

## Introduction

Chronic progressive renal fibrosis leading to end-stage renal failure in many patients with chronic kidney disease (CKD) is a global health care burden affecting billions of individuals worldwide 1, 2. Progressive tubulointerstitial fibrosis, the final common pathway leading to chronic renal failure in all kidney diseases, is associated with peritubular capillary (PTC) loss in both animal models and patients 3, 4. Thus, renal microvascular injury leading to PTC rarefaction and resulting in chronic renal tissue hypoxia is a typical feature of renal fibrosis 5. In this context, identifying new targets to delay renal fibrosis, including the preservation of PTCs, the clearance of apoptotic cells, and capillary regeneration, is of great interest.

Mesenchymal stem cells (MSCs) isolated from various extrarenal sources, including bone marrow, adipose tissue, and umbilical cord, are multipotent cells with robust self-renewal 6, regenerative 7, pro-angiogenic 8, and immunomodulatory properties 9, and multilineage differentiation potential 10 and can serve as ideal candidates for renal regenerative therapy. We previously demonstrated that human amniotic fluid-derived stem cells were capable of homing to PTCs after unilateral ureteral obstruction (UUO) -- a well-established *in vivo* model of tubulointerstitial scarring -- and alleviating renal interstitial fibrosis via increasing the renal microvascular density. Glial-derived neurotrophic factor (GDNF), an effective neurotrophic factor that protects nigral dopaminergic neurons, was first purified from B49 glial cells 11. We recently demonstrated the enhanced regenerative potential of human amniotic fluid-derived stem cells and their survival, differentiation, and secretion of regeneration factors following preconditioning with GDNF 12. Furthermore, GDNF-engineered human amniotic fluid-derived stem cells play a protective role in kidney injury 13. However, little is known about the detailed mechanisms underlying this process.

Analysis of the molecular mechanisms by which MSCs contribute to neovasculogenesis primarily through paracrine angiogenic activity indicated that secreted factors, such as exosomes, are key players to communicate with local and distant tissues 14,15. MSCs are avid producers of exosomes, 30-100 nm-sized small membranous particles of endosomal origin, which carry mRNA, microRNAs, and proteins, and exert protective effects by transferring the endogenous molecules and regulating apoptosis, inflammation, fibrosis, and angiogenesis in damaged cells 16-19. Indeed, the delivery of exosomes derived from MSCs has been shown to restore renal structure and function by an mRNA-dependent mechanism in experimental rodent models of acute renal failure 20. Choi *et al.* 21 showed that MSC-derived exosomes protected against the renal damage progression by ameliorating endothelial-to-mesenchymal transition and improving PTC rarefaction in UUO kidneys. However, whether GDNF mediates the paracrine actions of MSC-derived exosomes to preserve kidneys subjected to UUO injury has not been explored.

Sirtuin 1 (SIRT1), an (NAD^+^)-dependent deacetylase, exerts cytoprotective effects by inhibiting cell apoptosis, inflammation, and fibrosis 22. In the kidney, SIRT1 may inhibit renal cell apoptosis, inflammation, and fibrosis, which contribute to maintaining renal homeostasis, and their down-regulation leads to chronic and acute kidney diseases 23. Angiogenesis is one of these functions regulated by the interaction between SIRT1 and endothelial nitric oxide synthase (eNOS) 24. However, whether angiogenesis and one of its key regulators SIRT1 play a role in CKD development is unclear. Also, the mechanisms underlying the reno-protective effect associated with the exosomes derived from GDNF-modified human adipose mesenchymal stem cells (GDNF-AMSC-exos) remain to be defined.

In the present study, exosomes isolated from GFP-expressing adipose mesenchymal stem cells (GFP-AMSCs) and GDNF-modified human adipose mesenchymal stem cells (GDNF-AMSCs) (GFP-AMSC-exos and GDNF-AMSC-exos, respectively) were characterized by surface molecule expression. Moreover, we investigated whether GDNF-AMSC-exos had greater PTC-sparing and antifibrotic effects in the UUO mouse model than GFP-AMSC-exos. We further elucidated the mechanism underlying the effects of GDNF-AMSC-exos using a hypoxia/serum deprivation (H/SD) cell model *in vitro*, with a focus on SITR1/eNOS signaling.

## Materials and Methods

### Animals

One hundred and twenty athymic BALB/c nude mice (BALB/cJNju-Foxn1nu/Nju males, 4-6 weeks old) were purchased from Beijing Experimental Animal Research Center. All experiments involving animals followed the animal use protocol enacted by the Institutional Animal Care and Use Committee of Xuzhou Medical University (permit number: SYXK2015-0030). The mice were divided into the following four groups (30 mice in each group): sham sham-operated mice (sham group), UUO mice treated with phosphate-buffered saline (PBS) (UUO group), UUO mice treated with GFP-AMSC-exos (GFP-AMSC-exos group) and UUO mice treated with GDNF-AMSC-exos (GDNF-AMSC-exos group).

### Cell lines

Human adipose mesenchymal stem cells (AMSCs) were obtained from human adipose tissue samples and human umbilical vein endothelial cells (HUVECs) were purchased from ScienCell Research Laboratories, USA. Approval of all research involving human participants was obtained from the Institutional Review Board of the Affiliated Hospital of Xuzhou Medical University (permit number: xyfylw2013032). Human adipose tissues were acquired from lipoaspirate samples of abdominal fat from female donors (age range, 20-30 years) after obtaining informed consent.

### Preparation, culture, and identification of AMSCs

AMSCs were obtained from human adipose tissue samples according to classical methods reported in the literature and were grown in standard medium (DMEM/F12; Gibco, USA) containing 10% fetal bovine serum (FBS; Gibco, USA) at 37 °C and 5% CO_2_. The medium was replaced every 3 days throughout the entire culture period, and cells were split with 0.25% trypsin/0.02% EDTA at a ratio of 1:3 at each passage 25.

Cell immunophenotypes were analyzed by flow cytometry (Becton-Dickinson, USA). Briefly, 5×10^5^ AMSCs (within 3 passages) were collected by trypsinization and washed with PBS. Conjugated monoclonal antibodies PE-CD34 (#560941; BD Biosciences, USA), FITC-CD45 (#560976; BD Biosciences, USA), APC-HLA-DR (#560896; BD Biosciences, USA), PE-CD73 (#550257; BD Biosciences, USA), PE-Cy7-CD90 (#561558; BD Biosciences, USA) and APC-CD105 (#562408; BD Biosciences, USA) were used according to the manufacturer's instructions. Analysis of the fluorescence-activated cell sorting (FACS) data was carried out with FlowJo software version 10 (TreeStar, OR).

Osteogenesis and adipogenesis were examined to determine the multi-differentiation potential of AMSCs. For induction, 1×10^5^ AMSCs were seeded in 6-well plates and grown to 90% confluency, and the medium was then replaced with osteogenesis medium or adipogenesis medium (ThermoFisher, USA). Cells were fixed with 4% paraformaldehyde after 21 days and stained with alizarin red S or oil red O for observation under an optical microscope (Zeiss, Germany).

### AMSC transfection, selection, and GDNF expression

A green fluorescent protein (GFP) label for a lentiviral vector plasmid system carrying the GDNF gene was constructed by Shanghai Jikai Gene Technology Co., Ltd. AMSCs were transfected with lentiviral vectors at an appropriate multiplicity of infection (MOI = 20) according to the manufacturers' instructions. GFP expression was observed *via* fluorescence microscopy at 1, 3, and 5 days after lentiviral vector transfection. The expression of GDNF mRNA in AMSCs after GDNF transfer was verified by quantitative real-time PCR (qRT-PCR). The concentration of GDNF in both GDNF-AMSC-exos and GFP-AMSC-exos was measured by enzyme-linked immunosorbent assay (ELISA).

### Isolation and identification of GDNF-AMSC-derived exosomes

Exosomes were obtained from the supernatant of GDNF-AMSCs through ultracentrifugation according to classical methods reported in the literature 15. Transmission electron microscopy (TEM) and Western blotting for CD9, CD63, and CD81 were used to identify the collected exosomes as previously described. The size of the vesicles was determined by a dynamic light scattering technique using a Zetasizer Nano ZS analysis system (Zetasizer version 6.12; Malvern Instruments, UK).

### Mouse model of UUO and treatment of mice

The mice were housed in a specific pathogen-free animal facility under controlled environmental conditions at a temperature of 24 ± 1 °C, a humidity of 50 ± 10%, and a 12-hour light/dark cycle and water and food provided ad libitum. Laboratory mice standard feed was purchased from Jinan Pengyue Experimental Animal Breeding Co., Ltd. UUO was performed as previously described 26 *via* a surgical incision on the abdomen. One day after surgery, the mice were randomly divided into three experimental groups. GFP-AMSC-exos and GDNF-AMSC-exos were diluted with PBS to a final concentration of 1×10^3^ μg/mL, and the UUO mice were randomly treated with 200 μL of GFP-AMSC-exos, GDNF-AMSC-exos or PBS *via* tail vein injection using a 1 mL syringe. Mice in each group were sacrificed by sodium pentobarbital injection on day 7 after UUO surgery, and kidneys and blood samples for blood urea nitrogen (BUN) and serum creatinine (Scr) determination were collected. Kidney tissues were processed for histology, immunofluorescence, Western blotting and qRT-PCR.

For *in vivo* tracking, GDNF-AMSC-exos were labeled before delivery with green fluorescent PKH67. Mice were sacrificed 4 hours after tail vein injection. The localization of exosomes was evaluated in 7-μm UUO kidney sections by immunofluorescence staining with the exosome marker CD81. The secondary antibodies were conjugated to Alexa Fluor^TM^ 594 goat anti-rabbit IgG (H+L) (#A11037, 1:200; Invitrogen). At least 15 randomly selected fields were analyzed for colocalization of kidney and exosome markers by confocal microscopy (FV1000; Olympus, Tokyo, Japan).

### Histology

Kidneys were fixed in 4% paraformaldehyde for 24 hours, embedded in paraffin and sliced into sections (2-3 μm). Hematoxylin/eosin (HE) staining was used to evaluate pathological kidney injury, and Masson trichrome staining was carried out to estimate the extent of tubulointerstitial fibrosis 27.

### BUN and Scr measurement

Blood samples for the measurement of BUN and Scr were harvested through eyeball removal under anesthesia and centrifuged at 3000 rpm for 10 minutes to obtain mouse serum. BUN and Scr levels were investigated using colorimetric assays according to the manufacturer's instructions (Bioassay System, USA) 28.

### Fluorescence microangiography (FMA) and immunofluorescence analysis

According to the protocol published by Kramann *et al.* 29, all solutions were prewarmed to 41 °C before the procedure. The abdomen and thorax of mice were cut *via* a midline incision extending from the symphysis pubis to the jugulum after anesthetized with chloral hydrate (10.0%, 0.003 mL/g intraperitoneal). One milliliter of heparinized saline followed by 1 mL of 3 M KCl was injected into the beating left ventricle by intravenous infusion needle. The inferior vena cava was cut and 10 mL PBS was perfused, immediately followed by 5 mL of the agarose-microbead mixture (500 μL FluoSpheres plus 4.5 mL 1% agarose/mouse). Immediately after perfusion, kidneys were excised, and OCT-embedded organs were cryo-sectioned into 7 μm sections.

For immunofluorescence staining, sections were blocked in 10% normal goat serum and incubated with a primary antibody specific for CD31 (#14-0311, 1:100; eBioscience), SIRT1 (#13161-1-AP, 1:50; Proteintech) or α-smooth muscle actin (α-SMA) (#A2547, 1:200; Sigma-Aldrich) followed by an Alexa Fluor^TM^ 594 goat anti-rabbit IgG(H+L)-conjugated secondary antibody (#A11037, 1:200; Invitrogen). All images were obtained by confocal microscopy (FV1000; Olympus, Tokyo, Japan).

### H/SD *in vitro* and treatment

HUVECs were cultured in endothelial cell medium (ECM; ScienCell, USA) supplemented with 10% FBS (ScienCell, USA) and 1% penicillin/streptomycin. Cells were incubated in an incubator at 37 °C and 5% CO_2_. HUVECs were stimulated with H/SD as described previously. HUVECs cultured in serum-free ECM were exposed to hypoxia (94% N_2_, 5% CO_2_, and 1% O_2_) in an anaerobic system (Thermo Forma, USA) at 37 °C for 24 hours and treated with GFP-AMSC-exos (100 μg/mL), GDNF-AMSC-exos (100 μg/mL) or PBS at the onset of hypoxia. In the control group, HUVECs were maintained under normoxic conditions (95% air, 5% CO_2_) for equivalent periods 24.

### Cell apoptosis, migration, and Matrigel tube formation assays

Flow cytometry was used to assess membrane and nuclear events during apoptosis. HUVECs were suspended in 500 μL of binding buffer containing 5 μL of Annexin V-FITC and 5 μL of propidium iodide (PI) (KeyGEN, Nanjing, China), gently mixed with a pipette and incubated at room temperature for 15 minutes. The apoptosis rate was obtained from the percentage of double-stained cells by Annexin V and PI, as detected by flow cytometry (Becton-Dickinson, USA).

HUVEC migration was analyzed using 8.0-µm Transwell inserts (#3422; Corning, USA) as described previously. Cells (1×10^4^ cells per well; three replicates per group) were resuspended in serum-free ECM and plated into the upper chamber. A total of 500 μL of complete medium (containing 10% FBS) was added to the lower chamber. After incubation for 8 hours, cells inside each insert were removed with cotton swabs, and the migrated cells on the underside were stained with crystal violet solution for 10 minutes and counted in five random microscopic fields.

Tube formation was evaluated on Matrigel (#356234; Becton-Dickinson, USA) in a 48-well plate. In detail, 100 μL of cold Matrigel was transferred into each well of a 48-well plate and incubated at 37 °C for 30 minutes. A total of 5×10^4^ cells per well were dispensed onto the Matrigel. After incubation for 8 hours, tube formation was detected under an inverted microscope (Leica, Germany) and Image-Pro Plus 6.0 software was used to calculate the total tube length.

### SIRT1 siRNA and activation

Three SIRT1 siRNAs (siSIRT1 #1, #2 and #3) obtained from Biomics Biotechnologies Co., Ltd. (Suzhou, China) were used separately to knock down the expression of SIRT1 in HUVECs. Briefly, HUVECs were seeded into 60 mm dishes 24 hours before transfection and were then transiently transfected with 100 nM SIRT1 siRNA or control siRNA per 90% confluent dish using Lipofectamine 2000 (Invitrogen Life Technology, USA) according to the manufacturer's protocol. The inhibition efficiency of these siRNAs was verified by qRT-PCR, and the most effective siRNAs were used for downstream functional experiments. The same experiments were performed on cells treated with GDNF-AMSC-exos (100 μg/mL) or an equal PBS volume.

To assess whether SIRT1 activation can exert similar effects as GDNF-AMSC-exos on endothelial angiogenesis, HUVECs were plated at a density of 1.2×10^6^ cells/mL in 0.4 mL (0.5×10^6^ cells/treatment) in 24-well culture plates. Cells were preincubated with a SIRT1 activator (CAY10602; MCE, China) (50 μM) for 1 hour under general cell culture conditions.

### Immunoblotting

Homogenized renal tissues, cells, and exosome lysates were separated *via* 8% or 12% SDS-PAGE, transferred to nitrocellulose membranes (Millipore, Jaffrey, NH, USA), and probed with the following antibodies: anti-CD9 (#20597-1-AP, 1:500; Proteintech), anti-CD63 (#ab216130, 1:500; Abcam), anti-CD81 (#18250-1-AP, 1:500; Proteintech), anti-VEGF (#19003-1-AP; 1:1000, Proteintech), anti-HIF-1α (#AF1009, 1:1000; Affinity), anti-SIRT1 (#13161-1-AP, 1:500; Proteintech), anti-eNOS (#AF0096; 1:1000, Affinity), anti-p-eNOS (#AF3247, 1:1000; Affinity) and anti-β-actin (#4970, 1:1000; Cell Signaling Technology).

### Immunoprecipitation

A 50 μg sample of total HUVEC protein extract prepared for Western blot analyses was used for immunoprecipitation. Proteins were separated by SDS-PAGE and transferred onto PVDF membranes. Immunoprecipitated proteins were then detected with anti-eNOS antibody (#AF3247, 1:1000; Affinity).

### qRT-PCR analysis

Total RNA was extracted from renal tissues and cells using TRIzol reagent according to the manufacturer's instructions (Invitrogen, USA), and one microgram of RNA was reverse transcribed to first-strand cDNA using the GoScript reverse transcription system (Promega, USA). Quantitative PCR was conducted using SYBR master mix (Qiagen, Germany) on a Roche LightCycler 480II. Relative mRNA expression levels were calculated using the 2^-ΔΔCt^ method and were normalized to the corresponding expression levels of GAPDH. The primer sequences used to amplify the human and mouse RNA are shown in **Table 1**.

### Statistical analysis

Data are expressed as means ± SEMs. Statistical significance was assessed using Student's t-test or one-way ANOVA with Tukey's or Dunnett's post hoc tests or with a Kruskal-Wallis test for nonnormally distributed parameters. All statistical analyses, including linear regression analyses, were performed using GraphPad Prism software, version 5.0c. Values of *P* < 0.05 were deemed statistically significant.

## Results

### Characterization of AMSCs and exosomes

**Figure 1:**
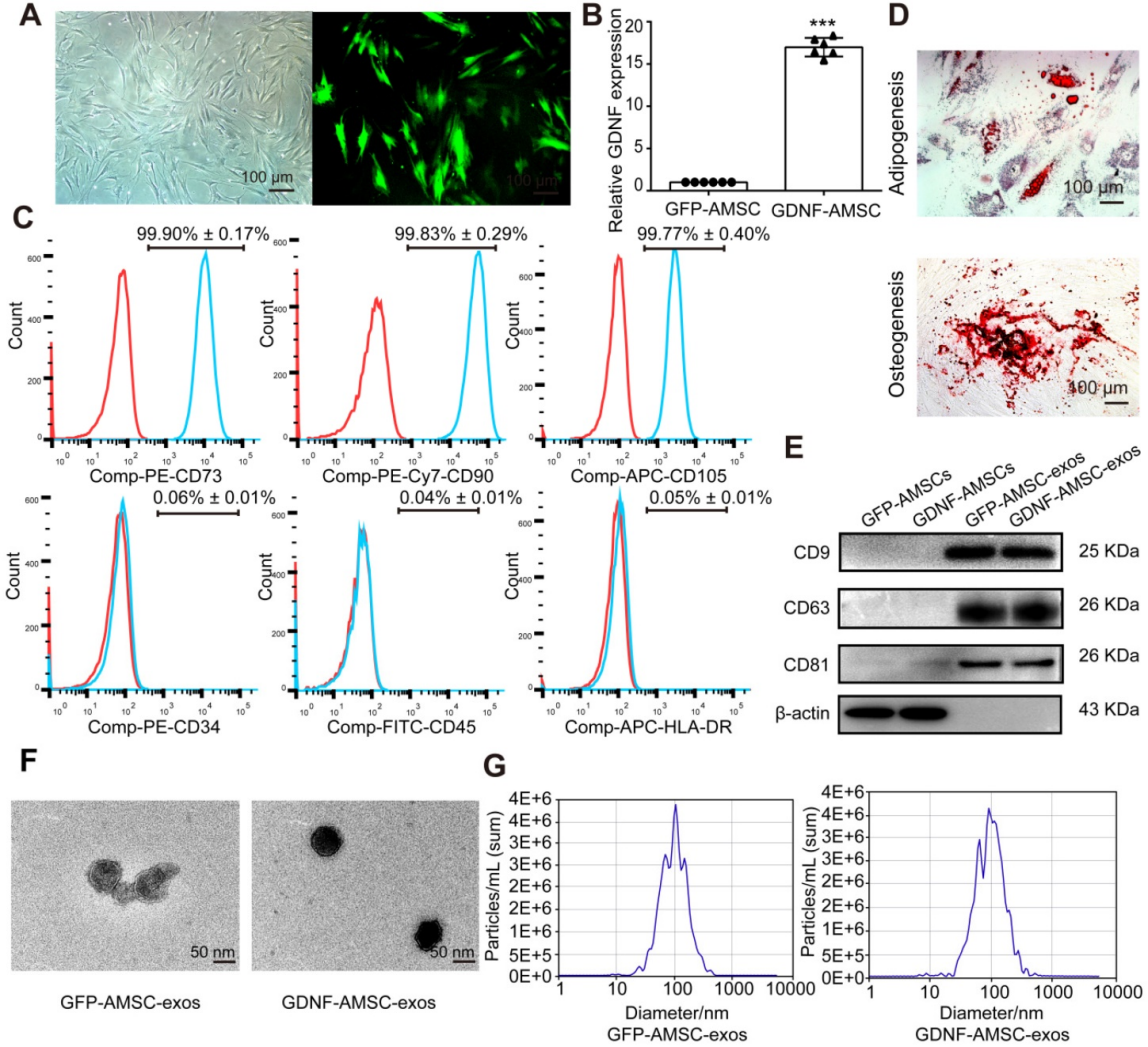
** Characterization of human adipose mesenchymal stem cells (AMSC)- and GDNF-transfected AMSCs (GDNF-AMSC)-derived exosomes. (A)** AMSCs were plastic-adherent, with fibroblastic morphology. Fluorescence expression of lentiviral vector in GDNF-transfected AMSCs at 72 hours. Approximately 70% of AMSCs expressed the GFP gene 72 hours after transfection, as indicated by the green fluorescence. Scale bar: 100 µm. **(B)** Quantitative real-time PCR (qRT-PCR) verification of upregulated GDNF mRNA expression in AMSCs after GDNF transfer. The results are shown as fold changes. ***P < 0.001 compared with controls. **(C)** Flow cytometric analysis of surface markers on AMSCs. **(D)** Representative images of AMSCs induced to differentiate into adipogenic and osteogenic lineages: (upper) oil red O staining for adipocytes; (lower) alizarin red S staining for osteocytes. **(E)** Representative Western blot images of CD9, CD63 and CD81 protein expression in the kidney cortex. β-Actin was used as the loading control. **(F)** Appearance of exosomes by transmission electron microscopy. Scale bar: 50 nm. **(G)** The size of the exosomes secreted from GFP-AMSCs and GDNF-AMSCs was measured by NanoSight analysis.

In this study, AMSCs were isolated from patients who underwent liposuction surgery and cultured in plastic flasks. These cells exhibited a fibroblast-like, spindle-shaped morphology (**Figure 1A**) and positively expressed CD73, CD90, and CD105 but negatively expressed CD34, CD45, and HLA-DR (**Figure 1C**). Positive oil red O staining or alizarin red S staining was observed after adipogenic or osteocytic induction of AMSCs for 21 days, which supported the cells' mesenchymal origin (**Figure 1D**).

AMSCs were transfected with lentiviral vectors expressing GDNF or GFP as the control (**Figure 1A**). The turbo GFP reporter gene was included in the constructs to visualize successful transduction. The transfection of GDNF showed high efficiency of GFP and GDNF expression by immunofluorescence microscopy and qRT-PCR analysis (**Figure 1B**). At 96 hours after transfection, exosomes were isolated from the GFP-AMSC and GDNF-AMSC supernatants. The two kinds of exosomes showed positive expression of exosomal markers such as CD9, CD63, and CD81 (**Figure 1E**). TEM demonstrated that the cells secreted substantial amounts of exosomes (**Figure 1F**). Nanosight analysis indicated the particle size distribution of the two kinds of purified exosomes to be between 30-150 nm (**Figure 1G**). Collectively, these data suggested that these nanoparticles were actually exosomes. ELISA results showed that the concentration of GDNF in GDNF-AMSC-exos was higher than in GFP-AMSC-exos (**Figure S1**).

### GDNF-AMSC-exos protected against UUO injury

**Figure 2:**
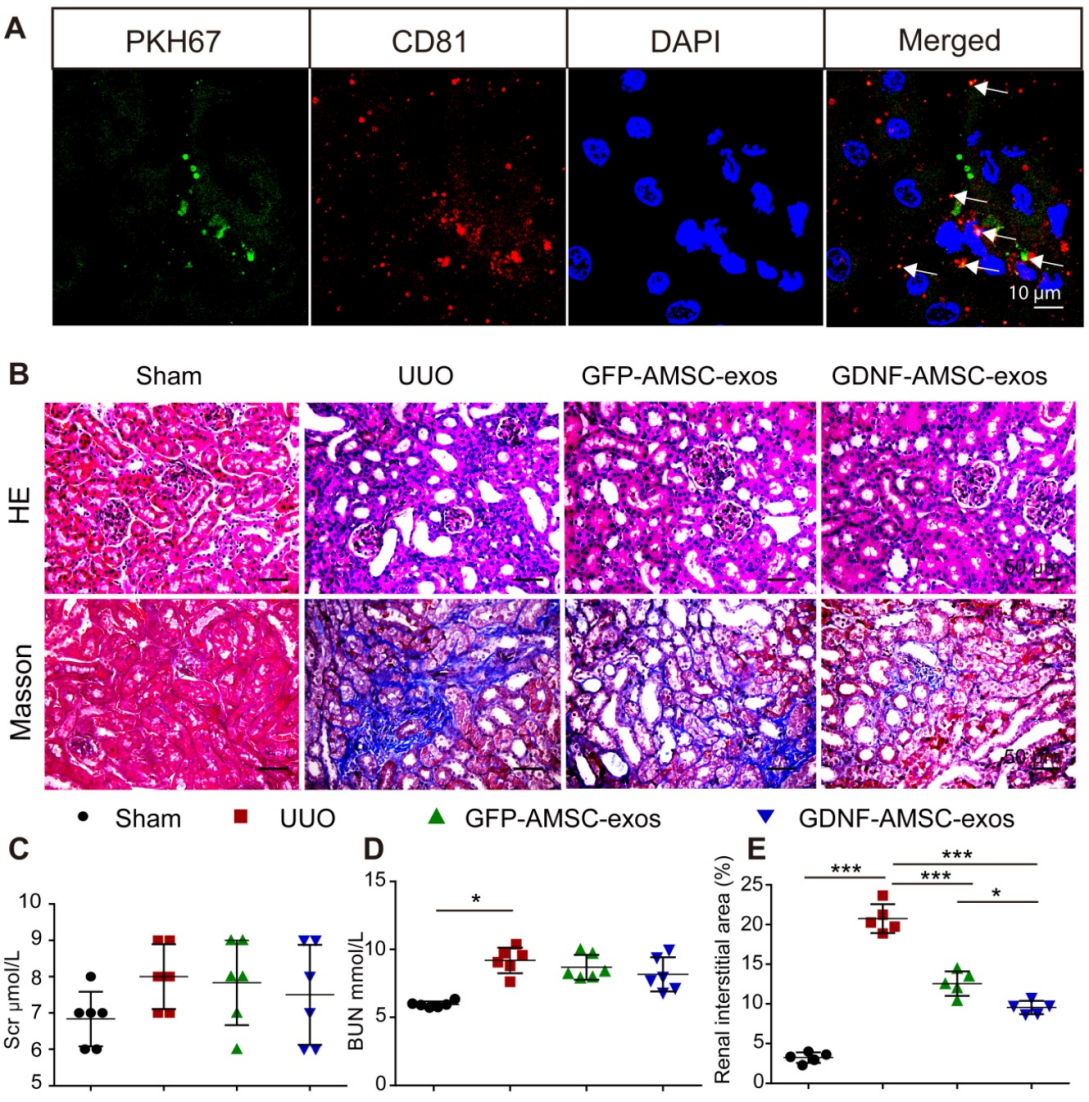
** Transplant of exosomes into unilateral ureteral obstruction (UUO)-treated kidneys contributed to the amelioration of renal injury. (A)** Fragments of green immunofluorescence-stained exosomes (PKH67, arrows) were detected in UUO kidneys 4 hours after administration, and only a fraction of the green fluorescence-stained exosomes were colocalized with CD81 (arrows). Green: PKH67, red: CD81, blue: DAPI. Scale bar: 10 µm. **(B)** Representative sections of kidneys treated with exosomes isolated from GFP-expressing adipose mesenchymal stem cells (GFP-AMSC-exos), exosomes derived from GDNF-modified human adipose mesenchymal stem cells (GDNF-AMSC-exos), or phosphate-buffered saline (PBS) 7 days after operation were stained with Hematoxylin/eosin (HE) and Masson trichrome to evaluate tubulointerstitial changes. Scale bar: 50 µm. **(C and D)** Renal function as evaluated by the serum creatinine (Scr) and blood urea nitrogen (BUN) levels in UUO mice treated with GFP-AMSC-exos, GDNF-AMSC-exos or PBS (n = 6). **(E)** Quantitative analysis of tubulointerstitial fibrosis in Masson trichrome-stained sections using the ImageJ program. Five randomly selected high-power fields were quantified and averaged to obtain the value for each mouse (n = 5 per experimental group). The results are expressed as the means ± SEMs of three different experiments. *P < 0.05; **P < 0.01; ***P < 0.001.

To evaluate the protective effects of GFP-AMSC-exos and GDNF-AMSC-exos, a mouse UUO model was generated, and the two kinds of exosomes were injected *via* the tail vein 1 day after surgery. The information on body weight, water, and food intake of mice were collected during experiments (**Figure S2A-C**). Seven days after UUO surgery, the body weight of the mice in the UUO group was significantly lower compared with the sham group (**Figure S2A**). We observed an increase in BUN (**Figure 2D**) associated with marked morphological damage, whereas mice subjected to sham surgery and injected with PBS alone displayed no histologic alterations. Injection of exosomes resulted in a decrease in tubular dilation relative to that in UUO mice treated with PBS (**Figure 2B**), but the BUN level (**Figure 2D**) did not significantly change in the group treated with GDNF-AMSC-exos or GFP-AMSC-exos. Also, the Scr level (**Figure 2C**) was not significantly altered in any of the groups.

We investigated the engraftment of GDNF-AMSC-exos in UUO kidneys. Green fluorescent PKH67-labeled exosomes were injected into BALB/c nude mice *via* the tail vein immediately after UUO, and mice were sacrificed 4 hours after the injection of GDNF-AMSC-exos. The green fluorescent PKH67-labeled exosomes were visualized in the UUO kidneys. To determine the nature of the green fluorescence signal, we performed immunofluorescence staining with antibodies against an exosome marker (CD81). Only a fraction of red-stained particles colocalized with CD81 (**Figure 2A**), suggesting that the green and red fluorescence signals were attributable to exosome fragments rather than intact exosomes retained within cells.

### GDNF-AMSC-exos ameliorated PTC rarefaction in UUO kidneys

**Figure 3:**
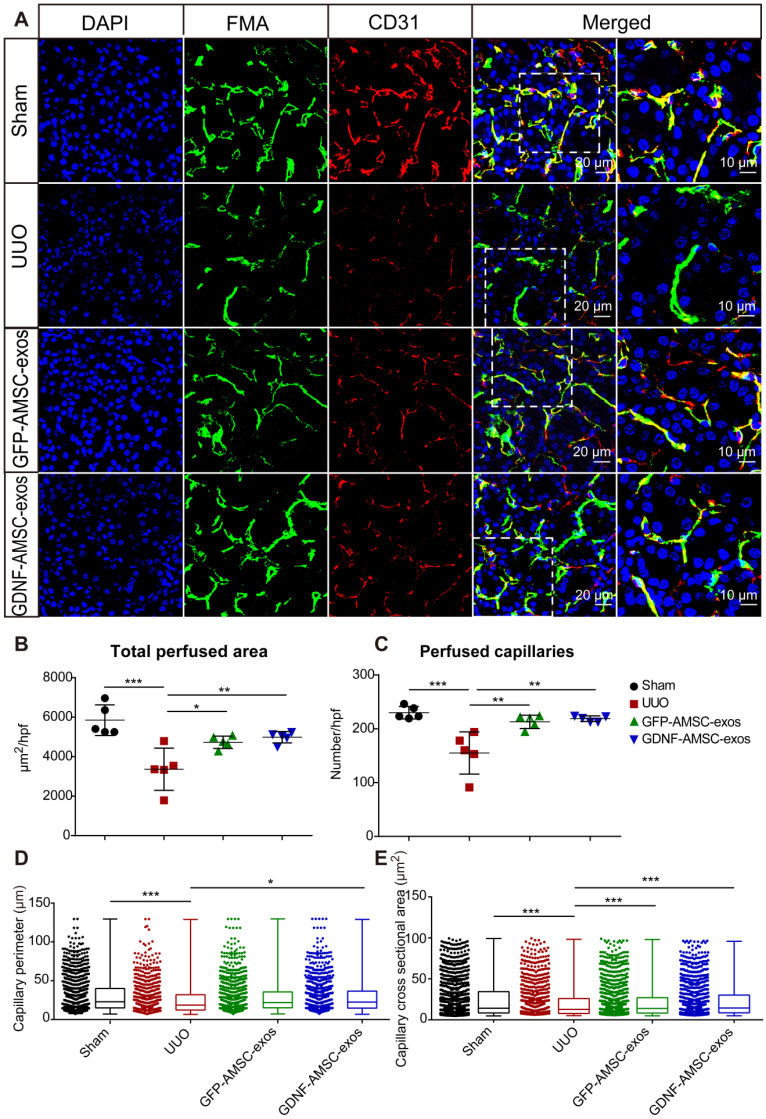
** High-throughput software-based analysis of fluorescence microangiography (FMA). (A)** FMA after unilateral ureteral obstruction (UUO) surgery in mice treated with exosomes isolated from GFP-expressing adipose mesenchymal stem cells (GFP-AMSC-exos), exosomes derived from GDNF-modified human adipose mesenchymal stem cells (GDNF-AMSC-exos), or phosphate-buffered saline (PBS), together with CD31 immunostaining, demonstrated capillary rarefaction in response to the severity of the injury. Capillaries with red CD31^+^ endothelial cells surrounding the green FMA solution; blue: DAPI. The scale bars represent 20 µm and 10 µm as noted. **(B and C)** Quantitative analysis of the total cortical cross-sectional capillary area and capillary number per high-power field. **(D and E)** Perimeter (mean ± SEMs, sham group: 30.76 ± 0.64 µm^2^; UUO group: 26.45 ± 0.72 µm^2^; GFP-AMSC-exos group: 28.94 ± 0.63 µm^2^; GDNF-AMSC-exos group: 29.85 ± 0.63 µm^2^) and cortical individual capillary cross-sectional area (mean ± SEMs, sham group: 25.47 ± 0.68 µm^2^; UUO group: 21.69 ± 0.75 µm^2^; GFP-AMSC-exos group: 22.27 ± 0.63 µm^2^; GDNF-AMSC-exos group: 23.13 ± 0.63 µm^2^) The data represent n = 5 mice per group. The mean ± SEMs are shown in B and C; box and whiskers indicating the 10th-90th percentiles are shown in D and E. *P < 0.05; **P < 0.01; ***P < 0.001.

According to our previous study, PTC density plays an important role in UUO injury. Here, we compared the effect of GFP-AMSC-exos and GDNF-AMSC-exos on PTC rarefaction following intravenous injection in UUO mice. We used FMA together with a MATLAB-based script to precisely and rapidly analyze the microvascular characteristics, including the single-capillary cross-sectional area and perimeter. All mice were subjected to the FMA procedure immediately before sacrifice 7 days after the UUO surgery. Confocal microscopy images of the kidneys demonstrated precise delineation of the PTC network by FMA (**Figure 3A**). The MATLAB-based analysis revealed a significant reduction of 41.6 ± 2.2% in the total cortical perfused area in the UUO group than in the sham group (**Figure 3B**) due to reductions in the capillary number, individual capillary cross-sectional area and perimeter (**Figure 3C-E**), indicating both a loss of capillary number and a reduction in the caliber of the remaining capillaries after UUO surgery.

Compared to the UUO group, both exosome-treated groups exhibited a significantly increased total perfused cortical area and capillary numbers, showing enhanced postinjury repair. Interestingly, the number of capillaries and the total perfused cortical area did not change significantly after treatment with GDNF-AMSC-exos compared with GFP-AMSC-exos, but there was a significant change in the perimeter. The most likely explanation for this observation is that angiogenesis in the capillaries may involve the intercalation or thinning of endothelial cells and the fusion of preexisting vessels allowing vessels to increase their length and GDNF-AMSC-exos had the ability to enhance angiogenesis. Comparison between FMA and CD31 staining demonstrated a larger reduction in the perfused FMA^+^ capillary area than in the CD31^+^ endothelial cell surface area, suggesting that some capillaries might lack perfusion following UUO. The group treated with GDNF-AMSC-exos showed significantly higher capillary density and perfusion than the UUO group by immunofluorescence, indicating their potential role in microvascular construction.

### GDNF-AMSC-exos ameliorated hypoxia and tubulointerstitial fibrosis in UUO kidneys

**Figure 4:**
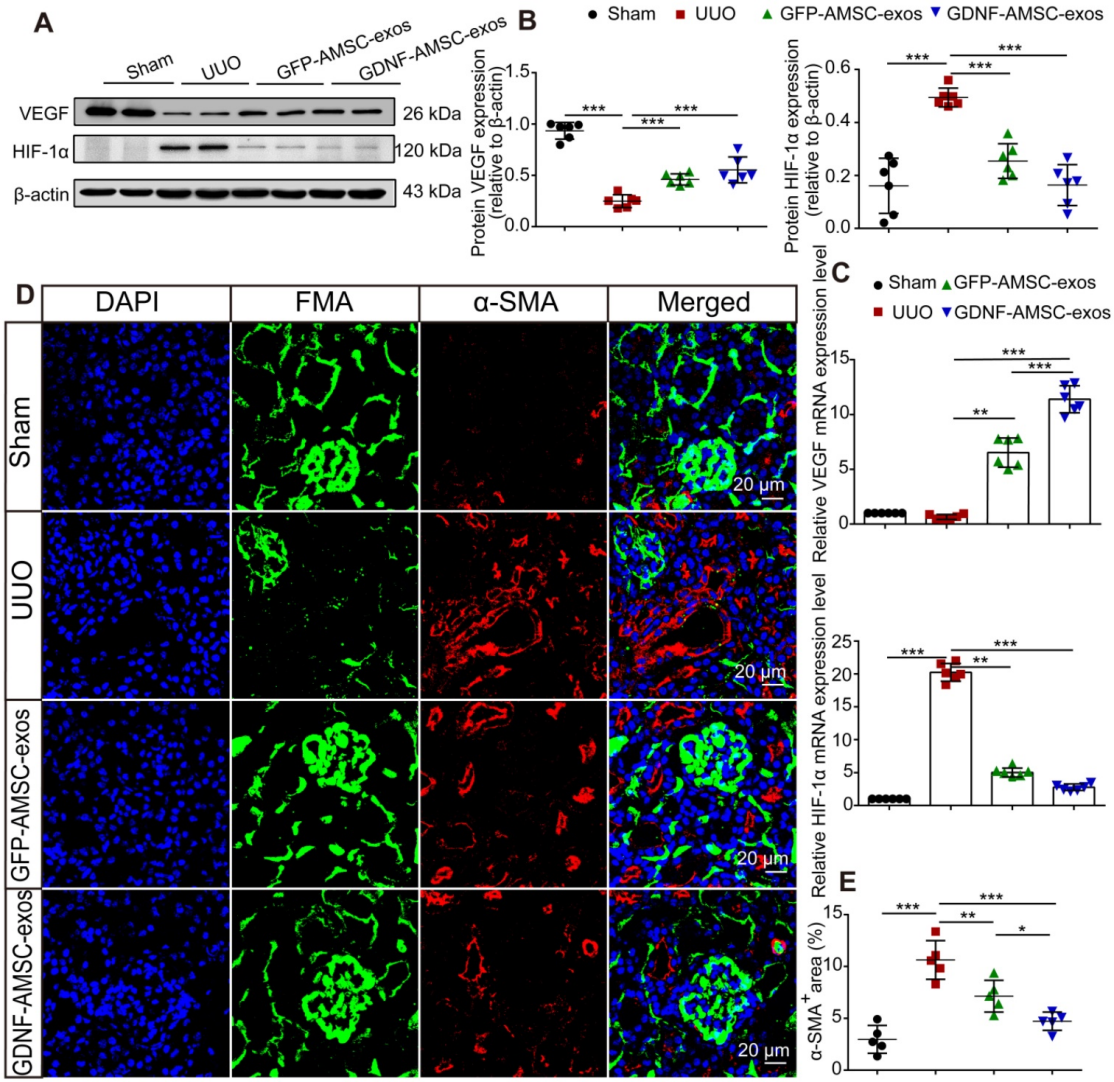
** Antihypoxic and antifibrotic effects of exosomes derived from GDNF-modified human adipose mesenchymal stem cells (GDNF-AMSC-exos) in UUO kidneys. (A and B)** Representative Western blot image of vascular endothelial growth factor (VEGF) and hypoxia-inducible factor-1α (HIF-1α) protein expression in the kidney cortex. β-Actin was used as the loading control. **(C)** Relative mRNA expression of VEGF and HIF-1α in the kidney cortex (n = 6). The scale bars represent 20 µm. **(D and E)** α-smooth muscle actin (α-SMA) staining and quantification revealed the induction of interstitial fibrosis. Green: fluorescence microangiography (FMA), red: α-SMA, blue: DAPI. The results are expressed as the means ± SEMs of three different experiments. *P < 0.05; **P < 0.01; ***P < 0.001.

It has been reported that hypoperfusion of PTCs induced chronic hypoxia, followed by progressive tubulointerstitial fibrosis 30. To verify that GDNF-AMSC-exos could ameliorate hypoxia in UUO kidneys by increasing PTC density, the expression of hypoxia-inducible factor-1α (HIF-1α), a key mediator of cellular responses to low oxygen, and its downstream mediator vascular endothelial growth factor (VEGF) were assessed 31. In the GDNF-AMSC-exo-treated group, VEGF expression was increased, and that of HIF-1α was reduced, suggesting that treatment with GDNF-AMSC-exos improved kidney hypoxia. This finding was confirmed by Western blotting (**Figure 4A, B**) and qRT-PCR (**Figure 4C**).

The possible antifibrotic effect of GDNF-AMSC-exos was examined in kidney sections stained with Masson trichrome in UUO kidneys treated with GFP-AMSC-exos and GDNF-AMSC-exos (**Figure 2B**). On day 7 after UUO injury, there were more fibrotic lesions in UUO kidneys injected with PBS control, whereas those injected with GFP-AMSC-exos or GDNF-AMSC-exos showed less fibrosis in tubuleinterstitial areas (12.6 ± 1.5% and 9.5 ± 0.8% versus 20.8 ± 1.8% of the total tubulointerstitial area, respectively, *P < 0.05) (**Figure 2E**).

As the activation of myofibroblasts is a key step in the development of renal fibrosis 32-34, we investigated the expression of proteins associated with myofibroblasts in UUO kidneys. Kidney sections immuno-stained for α-SMA revealed similar findings as those stained with Masson trichrome. Comparison between FMA and α-SMA staining following UUO demonstrated that mice injected with GFP-AMSC-exos or GDNF-AMSC-exos showed a larger perfused green FMA capillary area and a smaller α-SMA-stained myofibroblast surface area than mice injected with PBS control. Moreover, GDNF-AMSC-exos showed greater antifibrotic effects than GFP-AMSC-exos through increased PTC perfusion (**Figure 4D, E**).

### Activation of SIRT1/eNOS signaling pathway in UUO kidneys by GDNF-AMSC-exos

**Figure 5:**
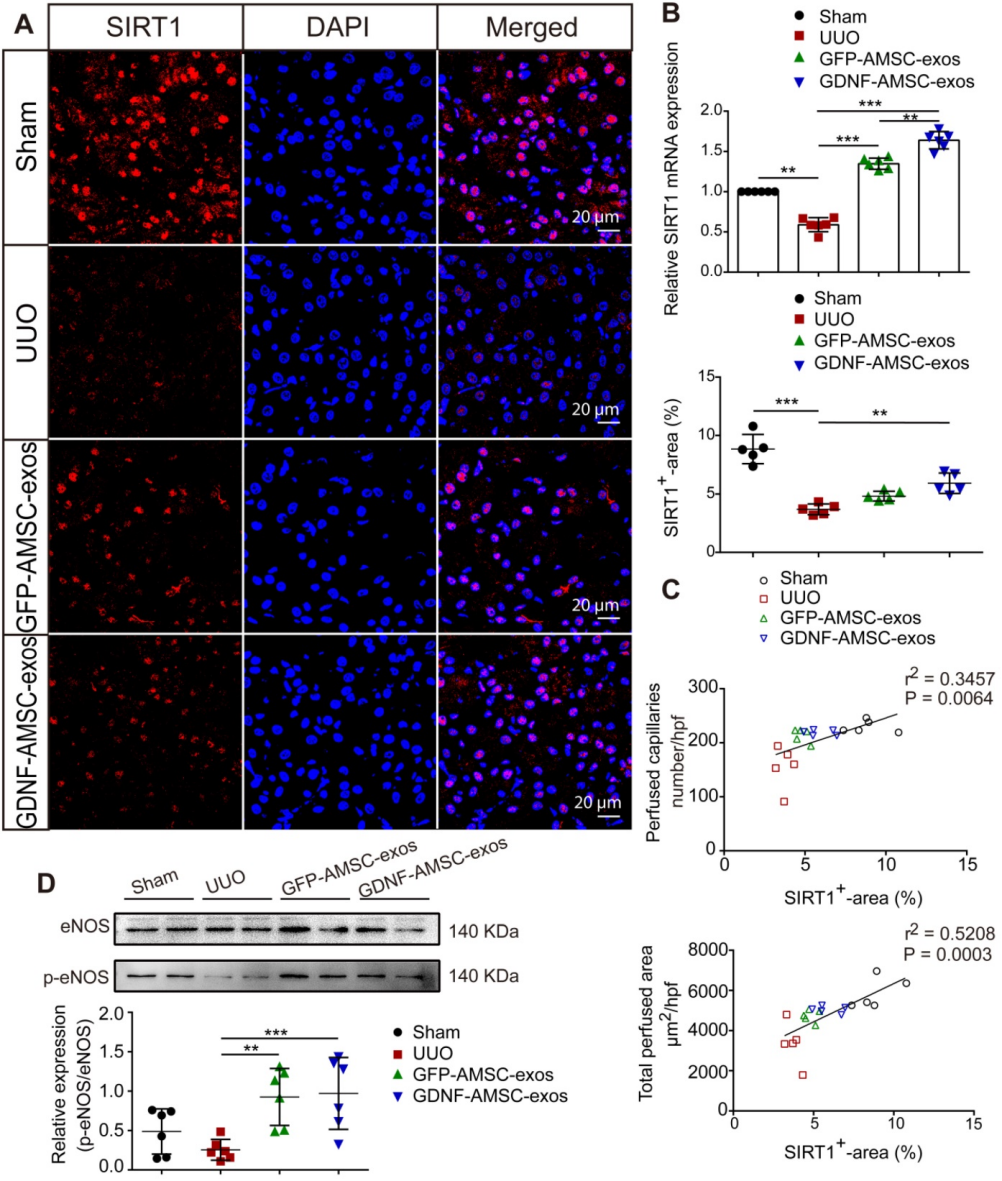
** The sirtuin-1 (SITR1) / endothelial nitric oxide synthase (eNOS) signaling pathway was activated in unilateral ureteral obstruction (UUO) kidneys after treatment with exosomes derived from GDNF-modified human adipose mesenchymal stem cells (GDNF-AMSC-exos). (A)** Co-staining of SIRT1 (red) and the nucleus (blue) was performed in kidney tissues. The scale bars represent 20 µm. **(B)** Relative mRNA expression of SIRT1 in the kidney cortex (n = 6). **(C)** The total capillary cross-sectional area (total perfused area, mm^2^/high-power field) and capillary number (number/high-power field) as assessed by fluorescence microangiography (FMA) showed a highly significant correlation with SIRT1 expression in the kidney. **(D)** The eNOS and phosphorylated eNOS (p-eNOS) levels in the kidney cortex were examined by Western blotting and quantified by densitometric analysis. The results are expressed as the means ± SEMs of three different experiments. *P < 0.05; **P < 0.01; ***P < 0.001.

SIRT1 has been shown to be a key regulator of vascular endothelial homeostasis, improving angiogenesis 22, 35, preventing renal dysfunction, and attenuating nephrosclerosis 36. Therefore, we examined SIRT1 after AMSC-derived exosome treatment. The qRT-PCR analysis showed that SIRT1 mRNA expression was decreased on day 7 of UUO injury but was increased after treatment with GFP-AMSC-exos or GDNF-AMSC-exos compared with the PBS control (**Figure 5B**). To examine SIRT1 response in the kidney, we conducted immunofluorescence labeling with antibodies against SIRT1. As shown in **Figure 5A**, SIRT1-positive tubular epithelial cells (TECs) were observed in kidneys, and their number decreased after UUO injury. Compared with the PBS control, treatment with GDNF-AMSC-exos increased SIRT1-positive TECs. Furthermore, there was a strong correlation between SIRT1 expression level in the kidney and a decrease in the total perfused peritubular cross-sectional area and PTC number (**Figure 5C**), suggesting that SIRT1 activation facilitates PTC construction. Since eNOS has been reported to be a downstream target of SIRT1 37, we hypothesized that the SIRT1/eNOS axis mediates the angiogenic response induced by GDNF-AMSC-exos. Western blotting revealed that treatment with GDNF-AMSC-exos significantly increased eNOS phosphorylation (**Figure 5D**). Thus, the activation of the SIRT1/eNOS pathway may be the underlying mechanism by which GDNF-AMSC-exos enhance PTC density to protect against renal fibrosis.

### *In vitro* anti-apoptotic, promigratory, and pro-tube formation effects of GDNF-AMSC-exos on hypoxic endothelial cells

**Figure 6:**
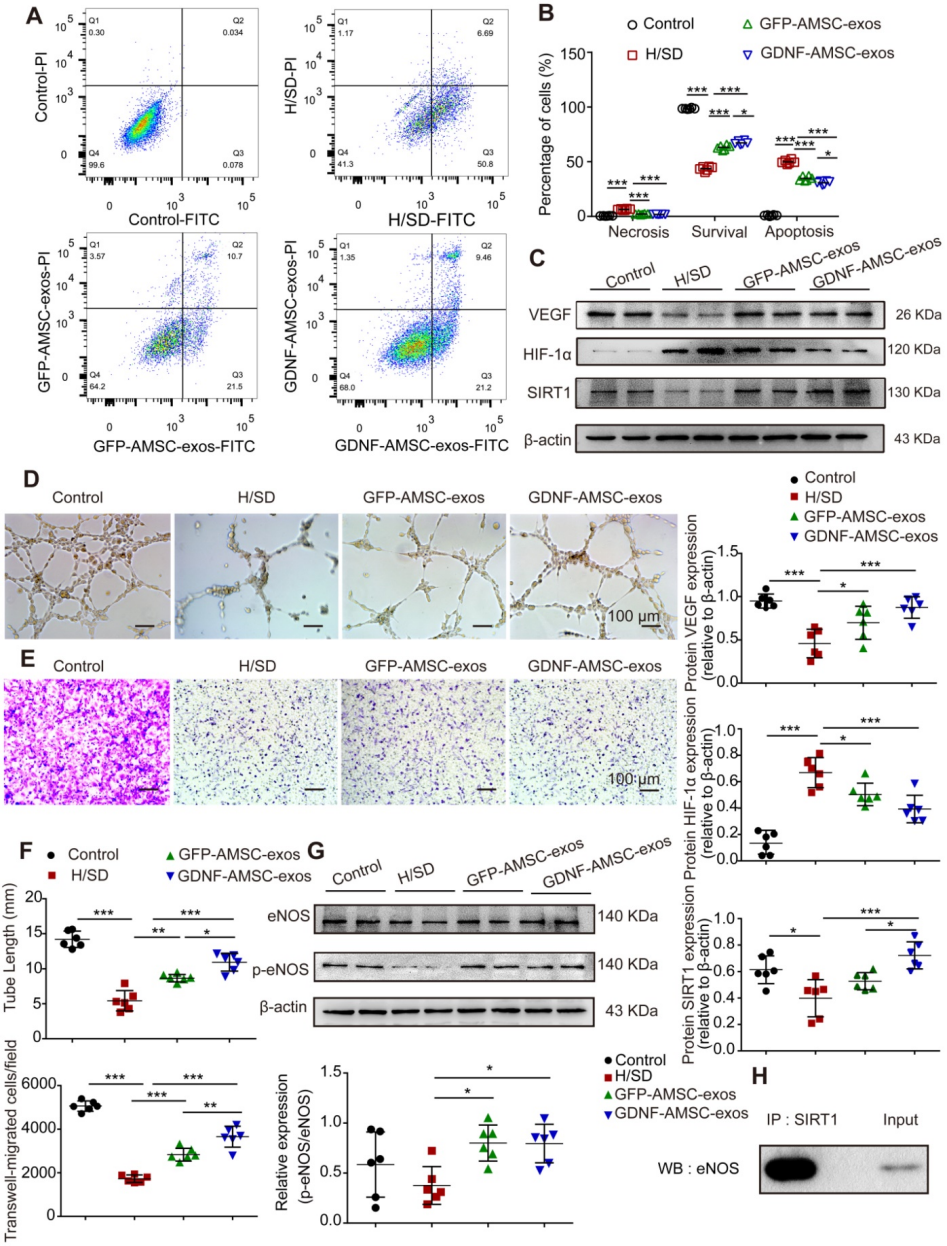
** Exosomes derived from GDNF-modified human adipose mesenchymal stem cells (GDNF-AMSC-exos) promote the migration and tube formation of endothelial cells subjected to hypoxia/serum deprivation (H/SD). (A and B)** Cell apoptosis was examined by Annexin V-FITC/propidium iodide (PI) staining using flow cytometry. **(C)** Representative Western blot image of vascular endothelial growth factor (VEGF) and hypoxia-inducible factor-1α (HIF-1α) protein expression in endothelial cells. β-actin was used as the loading control. **(D and F)** Representative images and quantification of human umbilical vein endothelial cell (HUVEC) tube formation in different treatment groups. Scale bar: 100 µm. **(E and F)** The migratory ability of HUVECs receiving different treatments was further confirmed by a Transwell assay. Scale bar: 100 µm. Quantitative analysis of the migrated cells in (D). N = 6 per group. **(G)** The levels of endothelial nitric oxide synthase (eNOS) and phosphorylated eNOS (p-eNOS) in HUVECs receiving different treatments were examined by Western blotting and quantified by densitometric analysis. β-Actin was used as the loading control. (H) Co-immunoprecipitation of endogenous eNOS and sirtuin-1 (SITR1) from HUVECs. Whole-cell lysates were immunoprecipitated with anti-SIRT1 antibodies. Immunoprecipitates were immunoblotted with anti-eNOS antibodies. The results are expressed as the means ± SEMs of three different experiments. *P < 0.05; **P < 0.01; ***P < 0.001.

Exosomes are known to act as vehicles to transport regulatory signals 14. To determine the effects of GFP-AMSC-exos and GDNF-AMSC-exos on endothelial cells with H/SD injury, HUVECs were incubated with both kinds of exosomes. After 24 hours of treatment, GDNF-AMSC-exos inhibited endothelial cell apoptosis more strongly than GFP-AMSC-exos, as indicated by the FACS analyses (**Figure 6A, B**). We determined the migration of endothelial cells after treatment with both exosomes. The results showed that although both types of AMSC-derived exosomes promoted endothelial cell migration, GDNF-AMSC-exos showed greater effects than GFP-AMSC-exos, as determined by the number of migrated cells in the Transwell assay (**Figure 6E, F**). Also, vascular formation ability was assessed by a Matrigel assay; GDNF-AMSC-exos increased the total tube length more than GFP-AMSC-exos, indicating a better pro-angiogenic ability of GDNF-AMSC-exos than GFP-AMSC-exos (**Figure 6D, F**). Overall, GDNF-AMSC-exos showed greater effects than GFP-AMSC-exos on the repair of endothelial injury-related processes, including anti-apoptotic and pro-angiogenic effects, indicating that exosomes derived from GDNF-AMSCs might contain different angiogenesis signals than exosomes from controls. Hence, both AMSC-derived exosomes were angiogenesis regulators, but GDNF-AMSC-exos showed greater effects.

### *In vitro* evidence of SIRT1 signaling protein expression in the repair of hypoxic endothelial cells by stimulation with GDNF-AMSC-exos

We hypothesized that SIRT1 mediated the effect of GDNF-AMSC-exos on the angiogenic response of hypoxic endothelial cells. To test this hypothesis, Western blotting was performed to assess the protein levels of VEGF, HIF-1α, SIRT1, eNOS and p-eNOS in HUVECs following treatment with GFP-AMSC-exos or GDNF-AMSC-exos under H/SD conditions for 24 hours. The data revealed that AMSC-derived exosomes induced a significant increase in the expression of VEGF and SIRT1 that was markedly decreased by HIF-1α (**Figure 6C**). AMSC-derived exosomes also induced a significant increase in the levels of eNOS and p-eNOS (**Figure 6G**). These data collectively suggested that AMSC-derived exosomes were positive regulators of HUVEC recovery from H/SD injury, although GDNF-AMSC-exos exhibited a stronger effect. Furthermore, co-immunoprecipitation assays showed an association between SIRT1 and eNOS in endothelial cells (Figure 6H). Thus, activation of SIRT1/eNOS pathway might be the underlying mechanism by which GDNF-AMSC-exos enhanced endothelial cell function.

**Figure 7:**
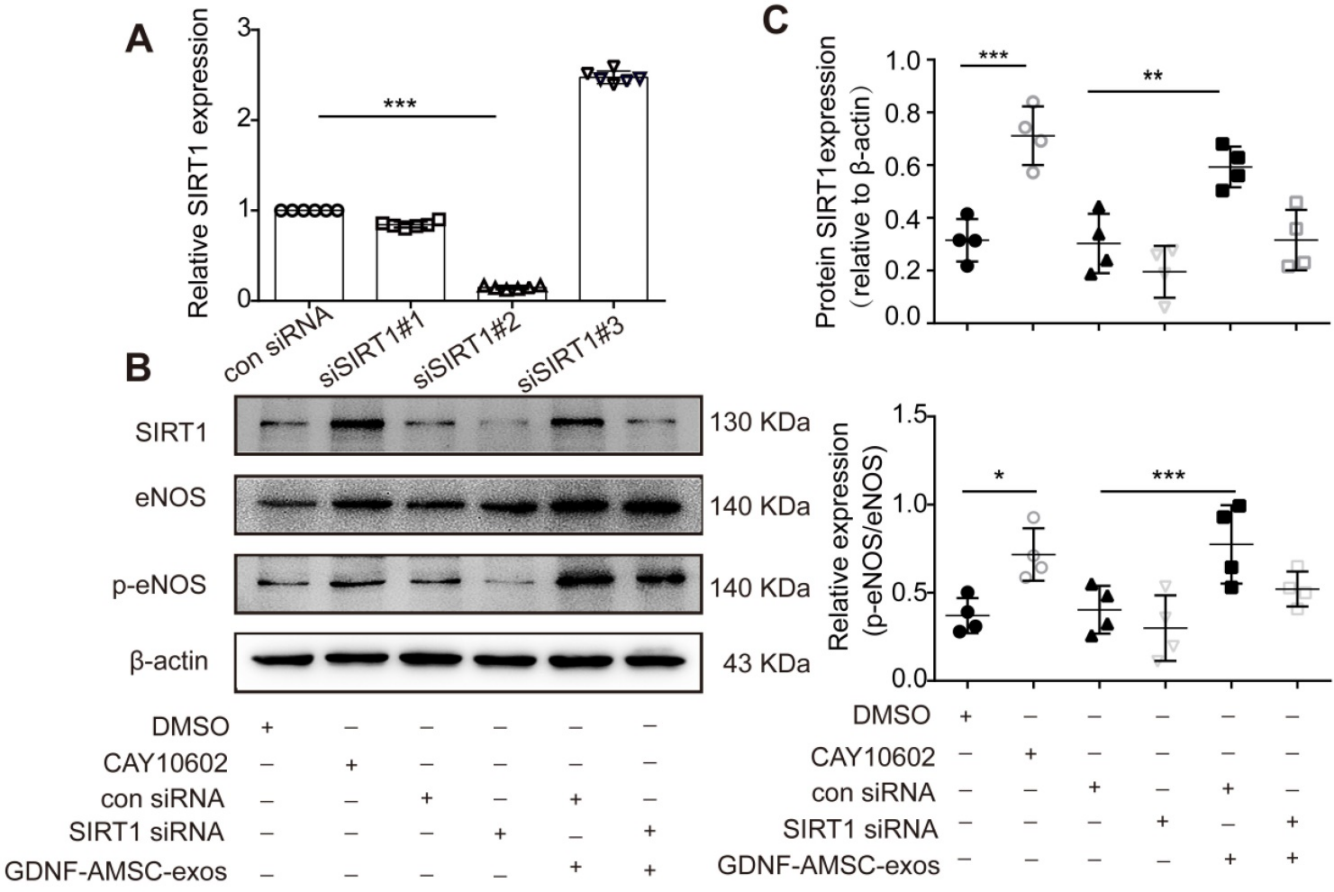
** The activation of sirtuin-1 (SITR1) / endothelial nitric oxide synthase (eNOS) signaling in human umbilical vein endothelial cells (HUVECs) subjected to hypoxia/serum deprivation (H/SD) in response to stimulation with exosomes derived from GDNF-modified human adipose mesenchymal stem cells (GDNF-AMSC-exos). (A)** The inhibition efficiency of the siRNAs targeting SIRT1 was verified by quantitative real-time PCR (qRT-PCR). N = 6 per group. **(B and C)** Western blot analysis of the SIRT1, eNOS, and phosphorylated eNOS (p-eNOS) protein levels in HUVECs treated with a SIRT1 activator (CAY10602) or vehicle control (DMSO), with SIRT1 siRNA or scrambled control (con siRNA) and with GDNF-AMSC-exos. β-actin served as internal control. The results are expressed as the means ± SEMs of three different experiments. *P < 0.05; **P < 0.01; ***P < 0.001.

To further verify that the SIRT1/eNOS pathway was involved in GDNF-AMSC-exo-mediated regulation, HUVECs were pretreated with a SIRT1 activator for 1 hour or transfected with SIRT1 siRNA before H/SD. Downregulation of SIRT1 was verified by qRT-PCR, and the most effective siRNA (siSIRT1 #2) was used in subsequent assays (**Figure 7A**). Our data showed that the SIRT1 activator increased but SIRT1 siRNA significantly decreased the level of p-eNOS (**Figure 7B, C**). Interestingly, treatment of SIRT1 siRNA HUVECs with GDNF-AMSC-exos achieved similar positive effects as treatment with the SIRT1 activator.

## Discussion

In the present study, we demonstrated that human AMSC-derived exosomes administered following UUO localized within kidneys and ameliorated PTC loss in tubulointerstitial fibrosis. Moreover, GDNF-AMSC-exos showed a much better capability for chronic kidney injury repair and angiogenesis than GFP-AMSC-exos in UUO mice. GDNF-AMSC-exos inhibited H/SD-induced apoptotic response in endothelial cells and promoted HUVEC migration and tube-like structure formation that was associated with the activation of the SIRT1/eNOS signaling pathway.

Cell-based regenerative therapy is being extensively evaluated as an alternative treatment modality for many patients with no other treatment options. Accumulating evidence indicates that MSCs attenuate renal injury and dysfunction in several animal models, and their efficacy in patients with renal disease is currently being tested in clinical trials 38, 39. We previously showed a reduced level of cell apoptosis in a hypoxia-reoxygenation model of GDNF-engineered amniotic fluid-derived stem cells cocultured with mouse renal tubular epithelial cells in a Transwell system 40. The current study extends our previous observations, demonstrating that UUO kidney structure and function are preserved 7 days after the delivery of GDNF-AMSC-exos. Therefore, this strategy may be useful, as exosomes circumvent concerns about extensive expansion, cryopreservation, complications, and mal-differentiation of live replicating MSCs. Eirin *et al.* 41 verified that intrarenal delivery of MSC microvesicles decreased renal inflammation, increased the number of reparative macrophages, and upregulated the expression of IL-10. Furthermore, microvesicles from MSCs have been reported to alleviate renal ischemic reperfusion injury and enhance angiogenesis in rats 42. Microvesicles, such as exosomes, secreted from MSCs exert protective effects by transferring endogenous molecules to salvage injured cells by regulating apoptosis 43, inflammation 41, fibrosis 44, and angiogenesis 45. However, the efficacy of these particles in chronic renal injury remains unclear.

Renal microvascular injury leading to PTC rarefaction and chronic tissue hypoxia is a major contributor to renal disease progression 46, 47. A common feature present in all kidney disease models is the reduction in PTC density, which is thought to fuel hypoxia and accelerate the development of interstitial fibrosis 48. To assess and compare the *in vivo* efficacy of GFP-AMSC- and GDNF-AMSC-derived exosomes, we employed a well-established *in vivo* model of UUO that replicates the disease conditions of human tubulointerstitial scarring. UUO is associated with rarefaction of PTCs, which are essential for providing nutrients and oxygen to the surrounding tubules and interstitial cells. Our study showed that GDNF-AMSC-derived exosomes stained with PKH67 engrafted in UUO kidneys. Notably, GDNF-AMSC-exos showed a much better capability for post hypoxic injury repair and angiogenesis than GFP-AMSC-exos. The results of our *in vivo* studies are consistent with this finding and show that in UUO kidneys, GDNF-AMSC-exos may have contributed to decreased intrarenal hypoxia and ameliorated inflammatory cell infiltration and subsequent tubuleinterstitial fibrosis. The internalization of GDNF-AMSC-derived exosomes by endothelial cells was accompanied by an attenuation of microvascular rarefaction, a major pathological characteristic of UUO that mediates many of its harmful sequelae. Furthermore, treatment of UUO mice with GDNF-AMSC-exos led to an improvement in kidney structure. So, we focused on angiogenesis as well as reduced fibrosis, which may be a subsequence of kidney repair in UUO by GDNF-AMSC-exos.

The involvement of exosomes in angiogenesis is not without precedents. Previous studies showed that exosomes derived from mesenchymal stem cells, including bone marrow, adipose tissue and umbilical cord, and pluripotent stem cells, can promote angiogenesis in ischemic conditions and attenuate tissue injury after an ischemic insult 49-51. Likewise, the potent angiogenic properties of exosomes were supported in our study by the active expression of VEGF in cell culture and UUO model. However, when the impact of each of the two groups of exosomes was evaluated individually, the results were slightly different, with GDNF-AMSC-exos showing greater effects. At odds, it is interesting that HIF-1α was down-regulated by GDNF-AMSC-exos, which means the elevation of VEGF was independent of the HIF-1α increase. We speculate that the reason might be GDNF-AMSC-exos modulated the HIF-1α regulators and changed the hypoxia microenvironment by transferring the endogenous molecules. Indeed, although the effect of GDNF-AMSC-exos was undoubtedly demonstrated *in vitro* and *in vivo*, it is likely that in an injury scenario, other mechanisms may also mediate the observed pro-angiogenic effects, namely through the modulation of oxidative stress and inflammatory response.

SIRT1 has been reported to be an early injury response signature within the kidney epithelium and to participate in the intrinsic mechanisms regulating kidney development and repair 36. A large body of literature has described the reno-protective effects of SIRT1 in acute kidney injury, mainly its effect on mitochondrial function 52, 53. He* et al.* used a SIRT1-deficient UUO mouse model, which, compared with their wild-type littermates, displayed increased susceptibility to oxidative stress and enhanced renal apoptosis and fibrosis 54. The authors confirmed that targeting SIRT1 with SIRT1 activators may also be a therapeutic strategy for minimizing or preventing renal damage resulting from increased oxidative stress. The current understanding of the role of SIRT1 in renal physiology and the pathogenesis of renal diseases is limited, as was recently summarized 23.

Previous investigations did not associate exosomes derived from MSCs with SIRT1 activation, both of which had been reported to facilitate regeneration of injured kidneys. Our study identified that intrarenal delivery of GDNF-AMSC-exos increased the number of PTCs, decreased renal fibrosis, and upregulated SIRT1 expression. Endothelial SIRT1 depletion or SIRT1 inactivation frequently accompanies many renal diseases. SIRT1 is highly expressed in endothelial cells, where it regulates numerous functions, including nitric oxide synthase production, cell senescence, and autophagy. In the vasculature, SIRT1 deficiency impedes angiogenesis. Vasko and colleagues confirmed that restoration of MMP-14 expression in SIRT1-depleted mice improved the angiogenic and matrilytic functions of the endothelium, prevented renal dysfunction, and attenuated nephrosclerosis 55. Similarly, our data established that SIRT1 was closely involved in the protective effects of GDNF-AMSC-exos against UUO and H/SD injury. SIRT1 suppressed apoptosis and stimulated angiogenesis by regulating the phosphorylation level of eNOS. However, the precise mechanism of action of SIRT1 remains to be elucidated.

Angiogenesis is rapidly initiated in response to hypoxic injury 56. The regulation of endothelial function is essential for maintaining neovascular homeostasis 57. eNOS is critical in the regulation of vascular function and can generate both nitric oxide (NO) and superoxide (O_2_^-^), which are key mediators of cellular signaling 58. Endothelium-derived NO, as an endogenous vasodilator, prevents vascular inflammation and thrombus formation by inhibiting platelet and leukocyte adherence 24. For example, vascular relaxation mediated by NO is a prerequisite for endothelial cell entry into the angiogenic cascade 59. Using SIRT1 siRNA interference, we further confirmed that the beneficial effects of GDNF-AMSC-exos on H/SD injury were SIRT1-dependent. The SIRT1 activator exerted beneficial effects on eNOS phosphorylation, thus protecting HUVECs from H/SD injury. Moreover, delivery of GDNF-AMSC-exos augmented the phosphorylation level of the eNOS protein, which was mimicked by treatment with the SIRT1 activator. However, inhibition of SIRT1 by SIRT1 siRNA decreased eNOS phosphorylation and weakened the protective effects of GDNF-AMSC-exos on HUVECs, suggesting that the phosphorylation of eNOS by SIRT1 is a promising strategy for combating H/SD injury in HUVECs.

Indeed, the significance of GDNF-AMSC-exos was clearly demonstrated *in vitro* and *in vivo*, and the observed antifibrotic effects are mediated through the modulation of PTC angiogenesis. Some limitations of this project should be acknowledged, however. It was reported that RNA overexpression in the source cell through lentiviral transduction usually results in enrichment of that RNA and the encoded protein in the exosomes generated by those cells 60. In this study, GDNF-overexpressing AMSCs released more GDNF protein through exosomes, compared with GFP-AMSCs. The change in total exosomal RNA content was proportional to the exosomal protein content. However, the effect of other RNAs in the exosomes could not be excluded. Due to the complexity of the exosomal contents, the SIRT1/eNOS pathway might be only one of multiple pathways that regulate angiogenesis. Residual proteins in the exosomes might also play a large role in exosome regulation. Clearly, further studies are required to unravel the possible diverse actions of exosomes derived from GDNF-AMSCs in CKD.

**Figure 8:**
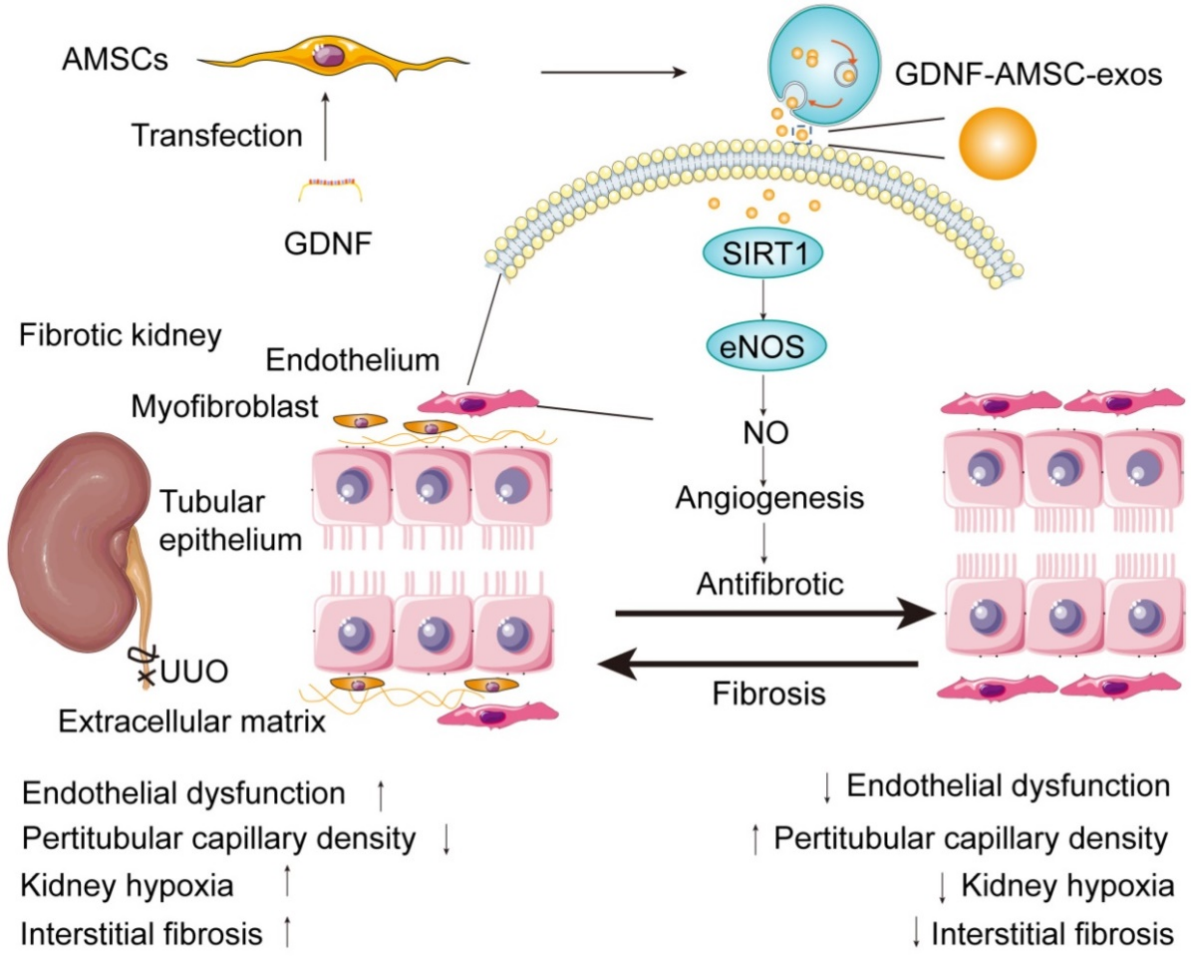
** Proposed mechanism by which exosomes derived from GDNF-modified human adipose mesenchymal stem cells (GDNF-AMSC-exos) ameliorate damage to unilateral ureteral obstruction (UUO)-treated kidneys by activating the SIRT1/eNOS signaling pathway.** In UUO-treated kidneys, the number and the size of peritubular capillaries (PTCs) were reduced and endothelial dysfunction occurred. Myofibroblasts and extracellular matrix deposition increased, and kidney hypoxia and renal fibrosis progressed. GDNF was transfected into human adipose mesenchymal stem cells (GDNF-AMSCs) *via* a lentiviral transfection system, and exosomes were obtained from the supernatants of GDNF-AMSCs through ultracentrifugation. GDNF-AMSC-exos were transplanted into UUO kidneys *via* tail vein injection and activated an angiogenesis program in surviving PTCs. GDNF-AMSC-exos enhanced sirtuin-1 (SIRT1) signaling, accompanied by increased phosphorylation of the endothelial nitric oxide synthase (p-eNOS) protein. GDNF-AMSC-exos prevented the reduction in the number of PTCs, maintained renal blood flow, and effectively suppressed the induction of myofibroblast progression. Thus, transplantation of GDNF-AMSC-exos can ameliorate kidney hypoxia and inhibit the progression of renal fibrosis.

In summary, our study identified the ability of exosomes isolated from GDNF-AMSCs in ameliorating renal fibrosis. Increased SIRT1 expression in the kidney and eNOS activation appeared to be the underlying mechanism that alleviated PTC loss and exerted potent protective effects (**Figure 8**). Furthermore, GDNF-AMSC-exos decreased apoptosis and enhanced the migration and angiogenic activities of endothelial cells *in vitro*. SIRT1 played a crucial role in the GDNF-AMSC-exo-dependent regulation of HUVEC H/SD injury and endothelial angiogenesis since the suppression of SIRT1 could markedly reduce the regulatory effects of GDNF-AMSC-exos. The current study is, therefore, an important step before the clinical translation of this approach. Our findings advance the current knowledge of angiogenesis in renal microvascular injury via the novel route of exosomes.

## Supplementary Material

Supplementary materials and methods, figures.

## Figures and Tables

**Table 1:** Primers used for quantitative reverse-transcriptase polymerase chain reaction

Genes	Forward primer (5'-3')	Reverse primer (5'-3')
Human-GDNF	5'-ACTGACTTGGGTCTGGGCTATG-3'	5'-TTTGTCACTCACCAGCCTTCTATTT-3'
Human-SIRT1	5'-CCCAGAACATAGACACGCTGGA-3'	5'-ATCAGCTGGGCACCTAGGACA-3'
Human-GAPDH	5'-ACAACTTTGGTATCGTGGAAGG-3'	5'-GCCATCACGCCACAGTTTC-3'
Mouse-SIRT1	5'-GGTTGACTTAGGTCTTGTCTG-3'	5'-CGTCCCTTGTAATGTTTCCC-3'
Mouse-GAPDH	5'-AGGTCGGTGTGAACGGATTTG-3'	5'-GGGGTCGTTGATGGCAACA-3'
Mouse-VEGF	5'-TATTCAGCGGACTCACCAGC-3'	5'-AACCAACCTCCTCAAACCGT-3'
Mouse-HIF-1α	5'-CGCCTCTGGACTTGTCTCTT-3'	5'-TCGACGTTCAGAACTCATCCT-3'

## Abbreviations

CKD: chronic kidney disease
GDNF: glial cell line derived neurotrophic factor
MSC: mesenchymal stem cells
AMSC: adipose mesenchymal stem cells
GFP: green fluorescent protein
GFP-AMSC: GFP-expressing adipose mesenchymal stem cells
GDNF-AMSC: GDNF-modified adipose mesenchymal stem cell
GFP-AMSC-exos: exosomes derived from GFP-expressing adipose mesenchymal stem cell
GDNF-AMSC-exos: exosomes derived from GDNF-modified adipose mesenchymal stem cell
PTC: peritubular capillary
UUO: unilateral ureteral obstruction
HUVEC: human umbilical vein endothelial cells
H/SD: hypoxia/serum deprivation
FMA: fluorescent microangiography
FACS: fluorescence-activated cell sorting
ELISA: enzyme-linked immunosorbent assay
TEM: transmission electron microscopy
PI: propidium iodide
HE: hematoxylin/eosin
qRT-PCR: quantitative real-time PCR
BUN: blood urea nitrogen
Scr: serum creatinin
FBS: fetal bovine serum
PBS: phosphate-buffered saline
SIRT1: Sirtuin 1
eNOS: endothelial nitric oxide synthase
p-eNOS: phosphorylated endothelial nitric oxide synthase
HIF-1α: hypoxia-inducible factor-1α
VEGF: vascular endothelial growth factor
α-SMA: α-smooth muscle actin
TECs: tubular epithelial cells
NO: nitric oxide
