# Supplementary material for: Exosomes derived from GDNF-modified human adipose mesenchymal stem cells ameliorate peritubular capillary loss in tubulointerstitial fibrosis by activating the SIRT1/eNOS signaling pathway
Source: Theranostics. 2020 Jul 25;10(20):9425–42. doi: 10.7150/thno.43315 (PMC7415791; doi:10.7150/thno.43315)
Supplement: Supplementary file 1 — Supplementary materials and methods, figures. [file thnov10p9425s1.pdf]

Supplementary materials and methods

Enzyme-linked immunosorbent assay (ELISA)

Exosomes were obtained from the supernatant of GFP-AMSCs and GDNF-AMSCs through ultracentrifugation and samples were diluted in PBS. GDNF levels were examined using the Elabscience ELISA kit (Elabscience, Wuhan, China) according to the manufacturer’s instruction manual. Absorbance was measured at 450 nm wavelength readings. The amount of GDNF in the sample was calculated from the standard curve.

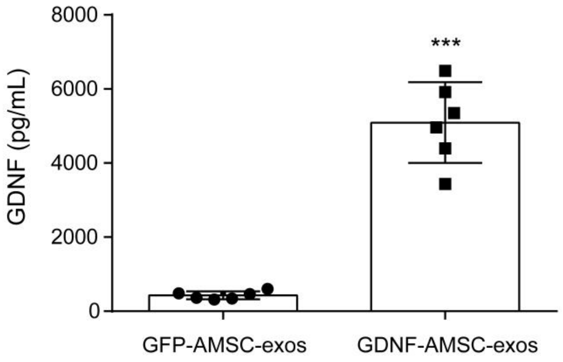

Figure S1. ELISA analysis of GDNF-AMSC-exos. ELISA verification of upregulated GDNF expression in exosomes derived from GDNF-modified human adipose mesenchymal stem cells. ***P < 0.001 compared with control.

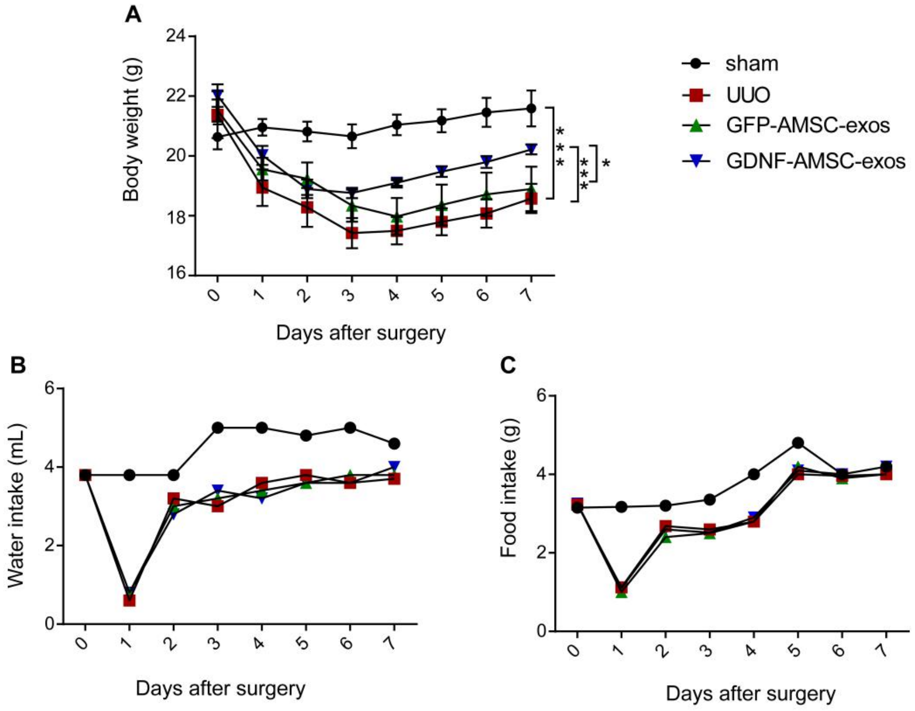

Figure S2. Effects of GDNF-AMSC-exos treatment on body weight gain, water and food intake in UUO-operated mice. (A) Differences in body weight among the four groups over time were analysed by two-way repeated measures ANOVA with Bonferroni's post-test. Values are expressed as mean ± SEMs (n= 6 per group), *$P < 0.05$, ***$P < 0.001$. **(B)** Differences in water intake among the four groups (average per mouse) over time were analyzed by Nonparametric Test with Kruskal-Wallis H. P=0.003. **(C)** Differences in food intake among the four groups (average per mouse) over time were analyzed by Nonparametric Test with Kruskal-Wallis H. $P = 0.354$.
